# Harnessing the power of RNA therapeutics in treating ischemic heart failure: the TRAIN-HEART story

**DOI:** 10.3389/fcvm.2023.1228160

**Published:** 2024-01-11

**Authors:** Alba M. Albert, Deepak Balamurali, Evangelia Beslika, Ileana D. Fernandez, Hussein H. Genedy, Reyhaneh Babaei Khorzoughi, Francesca Cecilia Lauta, Panagiotis Peppas, Isabella Ragone, Giorgia Rizzari, Marida Sansonetti, Joana Silva, Giulia Spanò, Kinga M. Wrona

**Affiliations:** ^1^ Cardiac Research, Regenerative Therapies, Target Discovery & Imaging, Miltenyi Biotec B.V. & Co. KG, Bergisch Gladbach, Germany; ^2^ Institute for Molecular and Translational Therapeutic Strategies (IMTTS), Hannover Medical School, Hannover, Germany; ^3^ Genetic Epidemiology and Statistical Genetics, Department of Biochemistry, CARIM School for Cardiovascular Diseases, Maastricht University, Maastricht, Netherlands; ^4^ Department of Cardiology, CARIM School for Cardiovascular Diseases, Faculty of Health Medicine and Life Sciences, Maastricht University, Maastricht, Netherlands; ^5^ Cardiovascular R&D Centre—UnIC@RISE, Department of Surgery and Physiology, Faculty of Medicine of the University of Porto, Porto, Portugal; ^6^ Institute of Pharmacology and Toxicology, Technical University of Munich, Munich, Germany; ^7^ Université Claude Bernard Lyon 1, UMR 5223, CNRS, INSA Lyon, Université Jean Monnet, Ingénierie des Matériaux Polymères, Villeurbanne, France; ^8^ King’s College London, British Heart Foundation Centre of Research Excellence, School of Cardiovascular and Metabolic Medicine & Sciences, London, United Kingdom; ^9^ IRCCS Humanitas Research Hospital, Rozzano, Italy; ^10^ Mirabilis Therapeutics BV, Maastricht, Netherlands; ^11^ Department of Experimental Pharmacology and Toxicology, University Medical Center Hamburg-Eppendorf, Hamburg, Germany

**Keywords:** ncRNA, heart failure, RNA therapeutics, cardiovascular diseases, nanomedicine, ITN, innovative training network, consortium

## Abstract

Ischemic heart disease (IHD) is one of the world's foremost killers, accounting for 16% of all deaths worldwide. IHD is the main cause of heart failure (HF), as it leads to pathological changes in the heart, improper pumping function and eventual death. Therapeutic interventions usually follow a systemic general strategy for all heart failure subtypes due to the lack of a deep understanding of the disease mechanisms. Hence, HF and IHD therapeutics need groundbreaking concepts to guide the development of a new therapeutics class that tackles the disease at a molecular level. The TRAIN-HEART consortium, a Marie Skłodowska-Curie Actions Innovative Training Network (MSCA-ITN) funded by the European Commission, was established with the goal of filling that gap and developing RNA-based cardiovascular therapeutics. Created in the context of the Horizon 2020 research and innovation program, TRAIN-HEART comprises three key work packages (WPs) focusing on the pathogenesis of heart disease (WP1), the therapeutic potential of RNA therapeutics (WP2), and the development of new efficient delivery systems (WP3). Fifteen international early stage researchers (ESRs) from multiple complementary scientific disciplines were recruited to collaborate with a network of PIs from nine academic and eight non-academic partners in various disciplines to fully harness their collective potential for the betterment of HF treatment. This article provides an overview of the benefits of being part of an MSCA-ITN, with its different training and networking opportunities, maximizing ESRs' potential and broadening collaborative research possibilities. Finally, it describes what was like to do a PhD during the COVID-19 pandemic, with all the uncertainty and concern attached to it. Luckily, TRAIN-HEART stood out as a proactive network, finding new initiatives and alternatives to promote scientific and personal development. By bringing together leading academic teams, (biotech) companies, and highly motivated researchers, TRAIN-HEART is expanding scientific horizons and accelerating future development of effective RNA-based therapies to treat IHD.

## 1 General Introduction

Cardiovascular Diseases (CVDs) continue to be the largest cause of mortality worldwide, with an estimated 17.9 million people dying as a result of it, accounting for 32% of global deaths (1). As of 2019, Europe recorded nearly 3.9 million deaths due to CVDs, with the related economic burden each year estimated to be around € 210 billion (2, 3). Despite numerous technological and scientific advances in recent decades, the management and treatment of CVDs, particularly Ischemic Heart Failure (IHF), remains predominantly generic, without taking into account other factors such as genetic variability between patients, age, gender, and disease subtypes. When the myocardial tissue is highly deoxygenated, it results in a loss of cardiomyocytes, which in turn triggers a series of events including formation of scar tissue, pressure overload and ventricular remodeling, eventually leading to death. Therefore, one of the most promising strategies to address this issue would be to restore the damaged heart muscle.

It was in 2014 that ITNs were born as “Innovative Training Networks” which had the aim to gather academic and non-academic organizations to train young stage researchers by doing science of the best quality with an innovation-approach (4). The TRAIN-HEART consortium is one such Innovative Training Network funded in 2019, in the context of Horizon 2020 research and innovation programme, with the aim of developing RNA-based therapeutics for the treatment of IHF (5, 6). For this purpose, leading academic teams and companies in the field have come together on the same platform to supervise Early Stage Researchers (ESRs) from various backgrounds. This dynamic and multidisciplinary group brings together different fields of science and medicine, providing an environment that facilitates new ideas and promotes diverse scientific research & training, with the common goal of pursuing novel discoveries in the field of Heart Failure (HF).

At TRAIN-HEART, the focus is three-pronged: first, to better understand the pathogenesis and the molecular mechanisms involved in the disease; secondly, to test novel microRNAs and study their role in cardiac regulatory pathways; and finally, to develop new strategies for drug delivery. During the project period, a team of fifteen ESRs are working on different yet inextricably linked PhD projects with this combination leading to an optimization of therapeutic efficacy.

## 2 Work package introductions

**Figure 1:**
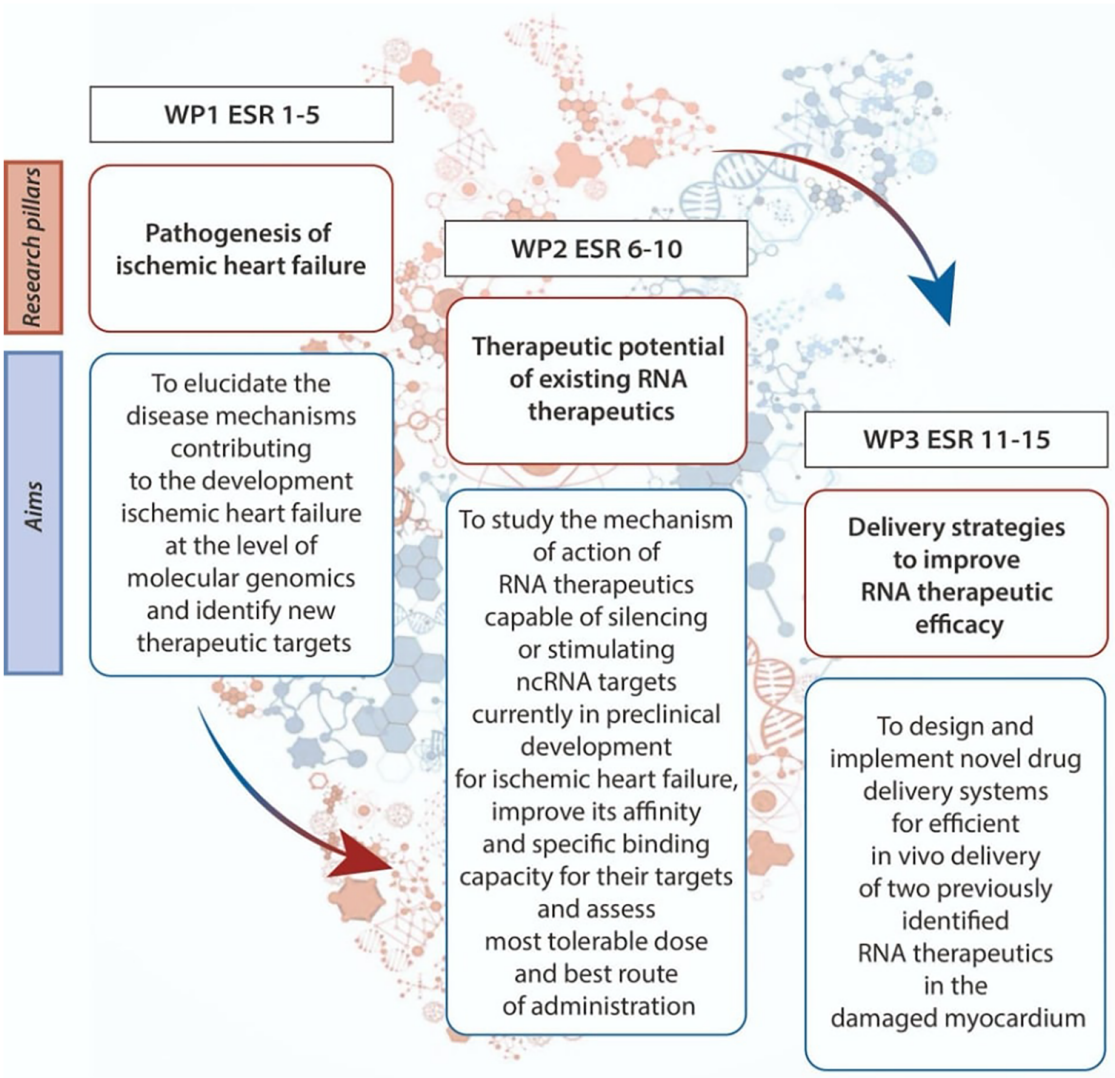
TRAIN-HEART working package groups. The organization and aims of 3 different working packages (WP) connecting 5 early-stage researchers (ESRs) each.

TRAIN-HEART brought together a group of academic and industry experts and fifteen promising ESRs (or research fellows) who had the final objective to explore and develop RNA therapeutics as a new class of pharmaceutical drugs to treat CVDs (Figure 1). These teams were spread all over Europe and the United Kingdom, enabling a multi-national collaborative network of researchers working towards a common goal.

To achieve this objective, TRAIN-HEART was organized into three work packages (WP) connecting five ESRs each, aiming to:

1. understand the pathogenesis of HF based on innovative high throughput technologies (WP 1)
2. study the therapeutic potential of existing RNA therapeutics (WP 2)
3. design novel drug delivery systems for RNA therapeutics (WP 3).

### 2.1 Work package 1 (WP1)

The WP1 team aimed to characterize expression patterns of pre-discovered non-coding RNAs (ncRNAs) and investigate their molecular and phenotypic effects in cardiac cells during HF progression. Each ESR's project focused on a specific topic designed to benefit from the established expertise of the partner institutions, remaining nevertheless complementary with the others. The WP members leveraged the use of high-throughput sequencing, microscopy and screening techniques and multi-species samples to create a repository of targets which would facilitate improved management of ischemic HF.

HF pathogenesis was investigated on multiple levels involving genomic and transcriptomic analyses on murine hypertrophic hearts in Humanitas Mirasole in Milan, Italy. Murine cardiac cell composition characterization in healthy and infarcted hearts was performed via advanced microscopy techniques at Miltenyi Biotech in Cologne, Germany. Proteomics analysis on a large cohort of human (ischemic & idiopathic) HF patients was carried out at King's College London in the United Kingdom, along with a targetome analysis of molecules involved in cardiac regeneration in neonatal rat cardiomyocytes. The team at Maastricht University, The Netherlands, worked towards the creation of a unified targetome database for novel and personalized therapeutics for ischemic HF, by collating information from genetic and epigenetic datasets and multiple genome-wide association studies (GWAS).

### 2.2 Work package 2 (WP2)

WP2 members mainly aimed to study the molecular mechanism of potential RNA therapeutics for cardiac regeneration and repair strategies for HF. Moreover, this team focused on improving the efficiency and efficacy of RNA therapeutics targeting dysregulated pathways in HF, assessing their best dose range(s) and route(s) of administration. Mirabilis Therapeutics, in The Netherlands, worked on the optimization of a class of RNA therapeutics: the antagomirs, which are engineered oligonucleotides used to silence dysregulated endogenous RNAs. Pre-clinical studies were performed in both small (mice) and large (pigs) animals to assess the efficiency and toxicity of antagomirs. Simultaneously, the team at the Technical University of Munich, Germany, studied the therapeutic effects of a promising small RNA inhibitor that silences an endogenous microRNA regulating cardiac fibrosis.

A team at Maastricht University, in The Netherlands, focused on post-transcriptional modified RNAs and their regulatory role in early human cardiac development. Finally, two keen research groups at Hannover Medical School and University Medical Center Hamburg-Eppendorf, in Germany, developed powerful *ex vivo* and *in vitro* models for cardiac diseases that can be used for future clinical translational studies. In particular, the ESR in Hannover further developed the multicellular Living Myocardial Slice (LMS) model into a pathological model by implanting cryoinjury. Similarly, the ESR in Hamburg created a multicellular model based on human iPSC-derived cell types. An Engineered Heart Tissue (EHT) was created by co-culturing five distinct cell populations (Cardiomyocytes, Fibroblasts, Vascular smooth muscle cells, Endothelial cells and Macrophages) all from the same cellular origin. This new EHT model represents a potential and promising human-like platform for investigating pathological cardiac hypertrophy. All together, these outstanding achievements will help to bridge from pre-clinical to clinical research.

### 2.3 Work package 3 (WP3)

The ultimate goal of WP3 was to pave the way to the optimisation of the delivery of ncRNA therapeutics in small and large animal models of HF. To address this aim, recent laboratory findings and technological innovations from TRAIN-HEART partners and collaborators were used. In particular, two novel types of cardiac-tissue-specific nanoparticles were constructed by the Claude Bernard Lyon University, France, and Humanitas Mirasole, in Italy,—biocompatible chitosan-based nanoparticles and negatively charged calcium phosphate nanoparticles respectively. Pharmacological and toxicological studies, including dose range determination and optimisation of the route of administration studies, were performed at the Technical University of Munich, Germany. Small and large animal models of HF and cardiac hypertrophy were developed at the University of Porto, Portugal, in order to investigate the efficacy and safety of these novel RNA therapeutics, targeting lncRNAs and microRNAs which were found to be differentially expressed in cardiac pathological conditions.

## 3 Benefits of being part of MSCA-ITN

### 3.1 Secondments

Most fellows had the exceptional opportunity to be seconded during their PhD programme, in order to ensure significant exposure to academic and/or industrial sectors. Secondment periods were very enjoyable parts of the fruitful experience of being a Marie Curie fellow. They were not limited to the respective WP Team that each fellow was assigned to, and they contributed to minimizing knowledge gaps and promoting social embedding in a new working environment. Given that there was a great variety in the educational and social backgrounds of the ESRs, it was very useful for them to be exposed to several new experimental techniques and different scientific approaches, while being in the partneŕs laboratory premises.

The main aim of the ESRs during their secondment period was to make proper use of laboratory techniques and research equipment, on which the host institution is specialized, in order to develop their PhD projects in several different directions. In addition, some fellows were glad to enjoy the multidisciplinary aspect of TRAIN-HEART ITN, by being able to learn and apply unique techniques and research methods, for the development of their personal career development plan and improving their future scientific career prospects, both inside and outside of academia.

### 3.2 Joint-trainings

Given the variety of backgrounds and knowledge of ESRs, a well-organized training program was planned. Several lectures were scheduled, by members of the consortium but also external professionals, to provide the ESRs with all the aspects of theoretical knowledge. Moreover, practical training and visits to some of the most innovative laboratories in Europe were organized to introduce the fellows to cutting-edge technologies and techniques (Table 1). This interactive environment provided an opportunity to broaden their research possibilities, create new ideas but also network and socialize while learning. Finally, important, non-scientific topics were not left aside. Aiming to the personal and professional development of the ESRs, these trainings were arranged to guide and advise them during their PhDs and also in their future career.

**Table 1:** TRAIN-HEART training program.

Institution	Training title
Mirabilis	Intellectual property and entrepreneurship for scientists (2 days)
Maastricht University	PhD/Postdoc course advanced optical microscopy
Hannover Medical School	Workshop: myocardial slice generation and functional assessment
King's College London	Proteomics & bioinformatics
Miltenyi (Bergisch Gladbach, DE)	Flow cytometry training
Hannover Medical School	Workshop: molecular assays to investigate telomerase activity
King's College London	High throughput screening course
Online	PhD, and next? Career options, skills and orientation for scientists

### 3.3 Network meetings & conferences

The ultimate goal of ITNs is to fulfil the huge demand for therapies and research entrepreneurship that the labor market is asking for. A good way of doing so is by giving young researchers the best tools and connections to start shaping their future towards this ultimate goal. We all know that scientific knowledge grows when shared and discussed, when forces are joined to row together. That is why TRAIN-HEART network meetings were established from the start. Each network meeting was treated as a real conference: they were held in the representative institutions, expert speakers were invited to give talks and the agenda included networking breaks and other social activities. Additionally, there was something special about those conferences; they offered trainings and lab visits, which allowed all the partners to get to know the facilities and potentiality of each research center. The oral speakers of the conference were, in this case, the young research fellows, who showed their last results in front of the network supervisors (all top researchers in the field), got their feedback, felt their enthusiasm, and listened to their ideas and suggestions.

**Figure 2:**
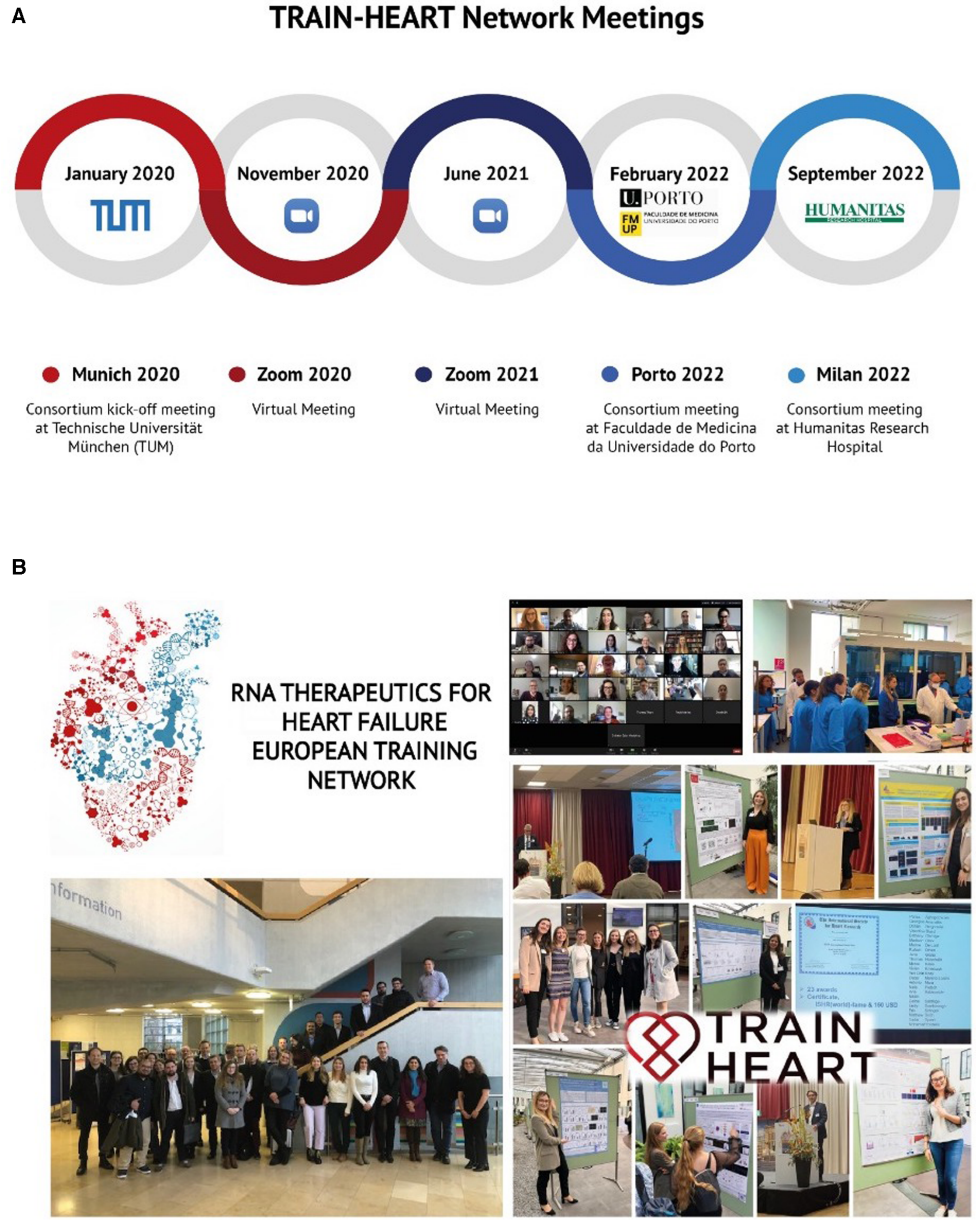
Benefits of being part of MSCA-ITN. (**A**) The main TRAIN-HEART network meetings. (**B**) MSCA-ITN training privileges.

Network meetings were the place to put together the individual pieces that conformed the 3 WPs and continue building up the puzzle we were all putting together (Figure 2). They were the time to look at all the projects and find synergies, points in common and solutions to each challenge.

## 4 The curveball that was COVID-19

### 4.1 Being a PhD candidate in the midst of a pandemic

COVID-19 pandemic marked a turning point in TRAIN-HEART networking purposes. We just had our first network meeting in Munich, when the entire world had to stop to face the biggest health hurdle of the 21st century. Being out of the laboratories for an indefinite amount of time meant to completely revise PhD projects and, sometimes, ambitions too. The original five-month secondments were reduced to shorter durations of just one or two months, trainings were suspended, and face-to-face network meetings were postponed indefinitely. Although our self-organized online Journal Clubs, Junior Board Meetings and Expert Lectures gave us the unique opportunity to fully dedicate ourselves to our education, remaining positive and proactive in this context was a real challenge. It was hard not to think about the quarantine time as a wasted time, and most of us started wondering whether they could still achieve good results during their doctorate.

### 4.2 How we moved virtual to cope with it (JC/expert lectures)

Originally, the TRAIN-HEART program included many meetings and conferences hosted at different institutions of the network. This meant not only meeting more than fifty people face-to-face but also traveling long distances across many country borders. Unfortunately, COVID-19 very soon became a burden for our consortium, resulting in a complete change of mindset. Networking had to take place somehow, and the only way to make it happen was by online meetings. Although offering us a means to stay connected, virtual meetings prevented the spontaneous social opportunities that come with chit-chatting with someone at the coffee machine, being hosted at the same hotel during a conference, not to mention the absence of social activities, pillars of most scientific meetings. We were missing a reunion as a source of collaborations and ideas, so we started a new trend: virtual Journal Clubs and Expert Lectures.

Every two weeks a paper published in a high-impact factor journal was presented and commented on together. This allowed us to learn, share ideas and get to know new scientific methods. Overall, TRAIN-HEART ESRs discussed more than 30 papers during the pandemic. However, we were still lacking the scientific knowledge coming from the experts that a real conference would have given us. Regardless of the hard times, we wanted to make the most out of the pandemic. Therefore, we started looking for those experts ourselves, sending them invitations to give us classes about their research and career expertise. By doing so, we attended no fewer than 20 lectures covering a broad range of topics: gene therapy applied to cardiac field research, ncRNAs of interest, *in vitro* assessment of cardiac function, nanoparticles, cardiomyopathies, state of the HF treatments, proteomics, development & application of new technologies for biomedical research & cellular therapeutics, high throughput screening for target discovery, target delivery approaches in CVDs, epigenetics, transcriptomics, microscopy, grant-writing, career development, etc.

## 5 What makes TH stand out?

### 5.1 New initiatives—junior board/dilemma game

As a part of the TRAIN-HEART program timeline, several educational initiatives were conceived for the benefit of the ESRs. From September 2020, ESRs organized monthly Junior Board Meetings (JBMs) to informally discuss with their peers their academic progress and the challenges associated with their respective research projects. At these meetings, there were also invited experts in the specific project's field to provide researchers with more technical and thoughtful feedback. Overall, JBMs turned out to be a vivacious environment, characterized by a genuine attitude to share experiences and scientific knowledge, making the initiative extremely useful for PhD candidates. In parallel, TRAIN-HEART ESRs arranged a few sessions of the “Dilemma Game”, a unique mobile application developed by the Erasmus University Rotterdam (EUR) (7). The students were presented with several dilemmas to encourage a discussion on relevant issues associated with research integrity, which represents a crucial hurdle in the work of a scientist.

### 5.2 Business case—elevator pitch

The TRAIN-HEART research program aims to produce various deliverables with high commercial value and to train its students to optimally benefit from these valuable results. Therefore, looking to expand the academic mindset of the PhD fellows to an entrepreneur attitude, TRAIN-HEART also included the “Intellectual Property and Entrepreneurship for Scientists” training, where the fellows were introduced to the basics of how to develop a business case and present the product to a jury of business experts.

Divided into the three WPs, the fellows had the assignment to design a new product to be introduced in the market. For this exercise, any intent of creativity was welcome, even beyond the research topic of the program. After six months of preparation, supported by comprehensive market research, intellectual property rights management, and a SWOT (Strength, Weakness, Opportunity, and Threat) analysis, as well as a reasonable realization and funding plan, the product was presented to the experts within an Elevator Pitch Competition. The winning team was awarded a travel grant to attend a conference or visit a research institute.

## 6 Wrap up

The TRAIN-HEART consortium, funded by the European Commission, aims to find novel treatment methods for HF and other CVDs based on RNA therapeutics. Considering the high relevance of the topic, TRAIN-HEART comprises top European Universities, well-known Principal Investigators, and extraordinarily motivated PhD students from various biological, medical, and non-medical backgrounds. The main focus of the consortium is to investigate the pathogenesis of HF through the use of innovative high throughput technologies, analyzing the therapeutic benefits of existing RNA-based therapies and developing novel drug delivery systems. Although within TRAIN-HEART, every PhD project is unique and original, a common goal stimulates fruitful collaboration among the participants.

In terms of improvement, ESRs could benefit from the many trainings, lab tours, secondments, and other educational activities. Some other aspects could also be of help for the long-term success of ITNs and all of them can be implemented by simple efforts from the ESRs themselves, during or after the training period—for example, creating an Alumni network for past participants would help foster collaborations and knowledge exchange even after the network closure. Similarly, including training on career development after completion of the PhD would give the ESRs an overview of their future opportunities in the scientific world. Finally, training on crisis management, problem-solving, and adaptability would give the ESRs some key skills that would be useful not only during their doctoral studies but also in their future positions.

As all good stories, TRAIN-HEART was also confronted with some difficulties; hard times came with the COVID pandemic. However, the ESRs proved that with motivation, ambition and eagerness to profit from any opportunity, great ideas can arise to circumvent the obstacles. This was, indeed, a big success. The numerous publications and conference presentations emanating from the collaborative work done as part of this consortium stand as proof (8). Therefore, there is no doubt that the environment provided by the ITN spurs cutting-edge research and long-lasting collaborations with partner sites within the consortium.
